# Hope Deferred

**Published:** 1891-03

**Authors:** 


### HOPE DEFERRED.

Again are we doomed to disappointment. Although the committee's bill to regulate the practice of medicine in Texas was introduced into the Senate about the middle of January, and was promptly acted upon by the Senate Committee on Public Health and recommended for passage, it has never advanced an inch beyond that point. The whole session of the Legislature appears to have been taken up with the railroad commission bill, which is still under discussion, and the school book bill, and a few others. The failure to get the medical bill pushed through was due perhaps, in a large measure, to the prolonged sickness of Chairman Cupples, and to the complete absorption of the time of the session with the above weighty matter. Many of the members have, in conversation with us, admitted that there is much need for some such bill; they appear to be impressed with the importance of the needs in this direction, and had the bill come before them for action, no doubt it would have been passed. At this writing, March 18, there is little prospect of reaching the bill, as the Legislature will abjourn April 6th, and its fate will have been decided before this is read by subscribers. Well, we can only wait and hope; medical legislation is rapidly gaining favor, and as there will likely be a called session of the Legislature, we will try it again. *Nil desperandum.*

